# Change in Publication Frequency in 2023

**DOI:** 10.3390/biomimetics8030292

**Published:** 2023-07-06

**Authors:** Stanislav N. Gorb

**Affiliations:** Department of Functional Morphology and Biomechanics, Institute of Zoology, Kiel University, Am Botanischen Garten 9, 24118 Kiel, Germany; sgorb@zoologie.uni-kiel.de

We are pleased to announce that starting in July 2023, *Biomimetics* will publish 12 monthly online issues. This change will facilitate expanded discoverability of the contents via a monthly Table of Contents and indexing in Web of Science, as well as the performance of other archival services in a timely manner. 

*Biomimetics* (ISSN 2313-7673) is an open access journal regarding biomimicry and bionics, dedicated to research that relates to the most basic aspects of living organisms and the transfer of their properties to human applications. The journal aims to provide a forum and a survey for researchers and professionals in the fields of materials science, mechanical engineering, nanotechnology, and biomedicine interested in exploiting biologically inspired designs in engineering systems, technology, and biomedicine that aim to develop novel solutions that enable sustainable innovation. 

Over the years, valuable contributions from authors and the participation of the many dedicated colleagues in the peer-review process have been vital to the continuing strength and success of *Biomimetics*. We sincerely appreciate all your contributions and look forward to your continued enthusiasm and support in the coming years.

